# Ten genes are considered as potential biomarkers for the diagnosis of dermatomyositis

**DOI:** 10.1371/journal.pone.0260511

**Published:** 2021-11-24

**Authors:** Lu Xiao, Wei Xiao, Shudian Lin

**Affiliations:** 1 Department of Rheumatology, Hainan general hospital (Hainan Affiliated Hospital of Hainan Medical University), Hainan, China; 2 Department of Respiratory, Hainan general hospital (Hainan Affiliated Hospital of Hainan Medical University), Hainan, China; University of Science and Technology Liaoning, CHINA

## Abstract

**Objective:**

This study aimed to identify the biomarkers and mechanisms for dermatomyositis (DM) progression at the transcriptome level through a combination of microarray and bioinformatic analyses.

**Method:**

Microarray datasets for skeletal muscle of DM and healthy control (HC) were downloaded from the Gene Expression Omnibus (GEO) database, and differentially expressed genes (DEGs) were identified by using GEO2R. Enrichment analyses were performed to understand the functions and enriched pathways of DEGs. A protein–protein interaction network was constructed to identify hub genes. The top 10 hub genes were validated by other GEO datasets. The diagnostic accuracy of the top 10 hub genes for DM was evaluated using the area under the curve of the receiver operating characteristic curve.

**Result:**

A total of 63 DEGs were identified between 10 DM samples and 9 HC samples. Gene Ontology and Kyoto Encyclopedia of Genes and Genomes enrichment analysis indicated that DEGs are mostly enriched in response to virus, defense response to virus, and type I interferon signaling pathway. 10 hub genes and 3 gene cluster modules were identified by Cytoscape. The identified hub genes were verified by GSE1551 and GSE11971 datasets and proven to be potential biomarkers for the diagnosis of DM.

**Conclusion:**

Our work identified 10 valuable genes as potential biomarkers for the diagnosis of DM and explored the potential underlying molecular mechanism of the disease.

## Introduction

Dermatomyositis (DM), a type of idiopathic inflammatory myopathy, is a rare autoimmune disease. It is characterized by muscle weakness and rash, and it is associated with high mortality rate [1]. In addition, DM is closely associated with an increased risk of malignancy compared with other types of myositis [2]. Given the rarity of the disease, the different characteristics of multiple disease subsets, and the lethal complications associated with it, the treatment for DM remains intractable.

Several factors may contribute to the pathogenesis of DM, including environmental factors and genetic susceptibility. The association between DM and various environmental triggers, such as Coxsackie B virus, enterovirus, sunlight, and some drugs, has been examined [3, 4]. Moreover, a genetic component may predispose individuals to DM. Immunological factors, including major histocompatibility complex, signal transducer and activator of transcription (STAT), autoantibodies, and cytokines, have been reported to be relevant to the development of DM [5–7].

Microarray techniques have increased the capability to detect the differentially expressed genes (DEGs) among different groups of people. Microarray techniques can demonstrate the expressed genes and identify special proteins produced by the gene, which were linked to altered biological processes in the progression of disease [8–10]. Therefore, using high-throughput genetic approaches, we are able to comprehensively understand the genetic architecture of DM. In the past, considerable important bioinformatic research has revealed the potential biomarkers in patients with DM. In 2018, Shuoshan Xie et al. discovered that IFITM2, LY6E, DDX58, and IFI6 were expressed at higher levels in the muscle tissue of patients with DM, indicating the important role of the interferon (IFN) signaling pathway in the pathogenesis of DM [11]. In 2020, one bioinformatic study has concluded that DEGs between DM and healthy control (HC) are primarily enriched in immune-related pathways [12]. Another bioinformatic study has revealed 10 hub genes, including ISG15, DDX58, IFIT3, CXCL10, and STAT1, and indicated that proinflammatory factors and IFN family proteins are highly associated with DM [13]. However, the exact pathogenesis of DM and the key pathogenic factor remain controversial. Hence, through the combination of microarray and bioinformatic analyses, the potential key genes and pathway networks that are closely related to the development of DM can be explored.

In this study, the microarray datasets, GSE48280 and GSE5370 for DM and HC, were first downloaded from the GEO database. 10 patients with DM and 9 HCs were included. Then, the data were integrated and reanalyzed. A total of 63 common DEGs were identified, including 61 upregulated genes and 2 downregulated genes, between DM and HC. DEGs were clustered with Gene Ontology (GO) and Kyoto Encyclopedia of Genes and Genomes (KEGG) pathway enrichment analysis. Furthermore, a protein–protein interaction (PPI) network was constructed using the online tool STRING, and Cytoscape was used to identify cluster modules and hub genes related to DM. Subsequently, we validated the 10 identified hub genes using two GEO datasets GSE1551 and GSE11971. The diagnostic accuracy of the identified hub genes for DM was evaluated with the area under the curve of the receiver operating characteristic curve (ROC–AUC).

This work provides insight into the mechanisms of the development of DM at the transcriptome level and explores potential biomarkers for diagnosis and treatment of DM.

## Materials and methods

### Data collection

**Fig 1:**
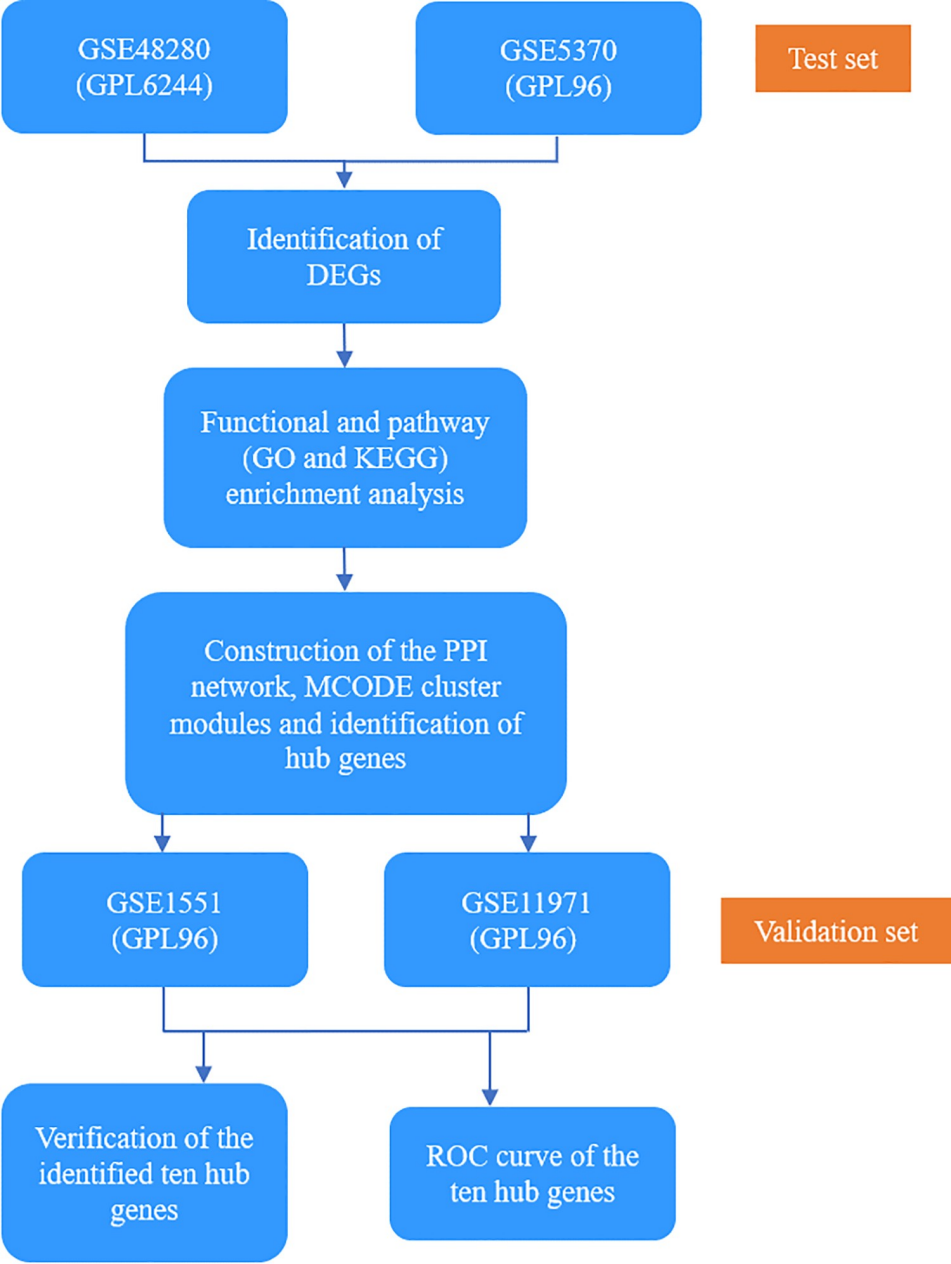
Flowchart of the study.

The GEO database is a public repository database, which stores a large number of gene functions and expressions [14]. We used ‘‘dermatomyositis” as the key word to search expression profiling of DM in the database. Finally, two datasets GSE48280 (GPL6244) and GSE5370(GPL96), including muscle biopsies from 10 patients with DM and 9 HCs, were selected as test sets [15]. Two datasets GSE1551 (GPL96) and GSE11971(GPL96), which included 32 DM samples and 14 HC samples were selected as validation sets [16] (Table 1). The overall flowchart of this study is shown in Fig 1.

**Table 1:** Information for selected microarray datasets.

GEO accession	Platform	Samples		Source tissue	Attribute
		DM	HC		
**GSE48280**	GPL6244	5	5	Human skeletal muscle	Test set
**GSE5370**	GPL96	5	4	Human skeletal muscle	Test set
**GSE1551**	GPL96	13	10	Human skeletal muscle	Validation set
**GSE11971**	GPL96	19	4	Human skeletal muscle	Validation set

### Identification of DEGs

The online web-based tool GEO2R was used to determine the DEGs of patients with DM. The row expression data of GSE48280 and GSE5730 were analyzed. Adjusted P value< 0.05 and |log2 fold change|>1 were set as the threshold and considered significant between the gene expression difference to identify the DEGs between patients with DM and HCs. Online tool Draw Venn Diagram (http://bioinformatics.psb.ugent.be/webtools/Venn/) was used to detect the overlapping DEGs among the two datasets.

### Functional and pathway enrichment analysis

R packages (clusterProfile and ggplot2) were used to perform GO enrichment and KEGG pathway analysis for the identified DEGs [17]. ClusterProfile package was used for analysis, and ggplot2 was applied to visualize the results.

### Construction of the PPI network

A PPI network for the DEGs was constructed using the STRING database (http://www.string-db.org/) which is an online tool to identify and predict interactions between genes and proteins (the cut-off standard as a combined score >0.4) [18]. Then, Cytoscape was used to visualize the results. Molecular Complex Detection (MCODE) V1.5.1, a plug-in of Cytoscape, was applied to identify significant modules (MCODE score ≥4) [19]. Moreover, Cytohubba, which is another plug-in of Cytoscape, was used to study essential nodes in the network. The nodes with high degrees of interaction were considered as hub genes [20].

### Statistical analysis

Statistical analysis was performed using Rstudio. The expression levels of 10 identified hub genes were validated by GSE1551 and GSE11971 using an independent sample *t* test. The ROC-AUC was performed by GraphPad PRISM 7. It was used to compare the diagnostic performance of different hub genes. The different expression levels of the hub genes were searched in the Series matrix file of GSE1551 and GSE11971. P < 0.05 was considered statistically significant.

## Results

### Identification of DEGs

**Fig 2:**
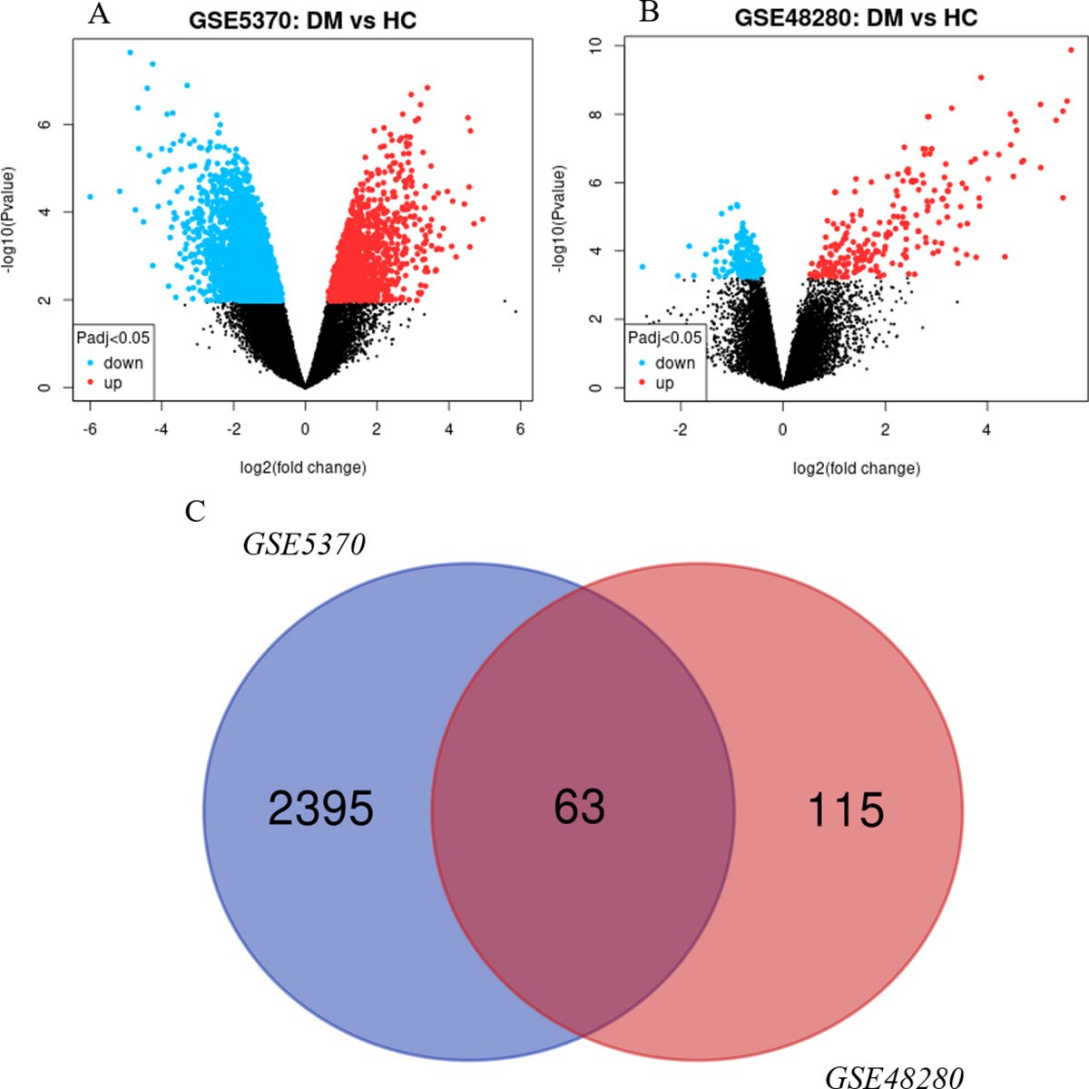
Identification of DEGs. (A) Volcano plot of GSE5370, (B) Volcano plot of GSE48280, and (C) Venn diagram of common DEGs from the two datasets. Data points in red and blue represent upregulated and downregulated genes, respectively.

Two gene expression datasets containing patients with DM and HCs were analyzed with two-group comparisons (P < 0.01), and then 2458 and 178 DEGs were identified from GSE48280 and GSE5370 respectively. The volcano plots of the two gene datasets are shown in Fig 2A and 2B. After integration of these DEGs by using bioinformatic analysis, a total of 63 common DEGs were identified in patients with DM (Fig 2C), including 61 upregulated genes and 2 downregulated genes.

### Functional enrichment analysis of DEGs

**Fig 3:**
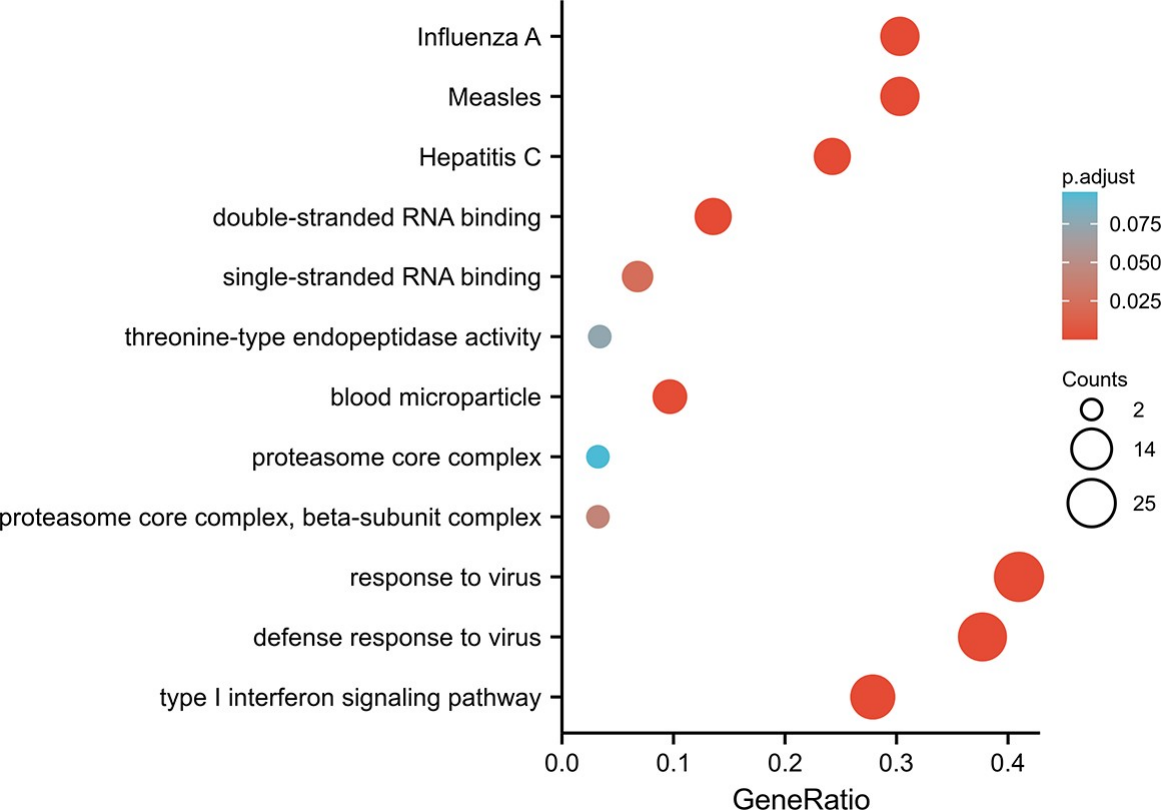
GO enrichment and KEGG pathway analysis of the overlapping 63 DEGs.

The 63 overlapping DEGs were analyzed using GO and KEGG enrichment (Fig 3). Based on GO enrichment, the biological process acts primarily on influenza A, measles, and hepatitis C. These proteins are primarily located in blood microparticle, proteasome core complex, and proteasome core complex, beta subunit complex. With regard to molecular functions, these proteins play a role in double-stranded RNA binding, single-stranded RNA binding, and threonine-type endopeptidase activity. KEGG pathway analysis presents that these proteins are primarily involved in response to virus, defense response to virus, and type I IFN signaling pathway.

### PPI network analysis, MCODE cluster modules and hub gene identification

**Fig 4:**
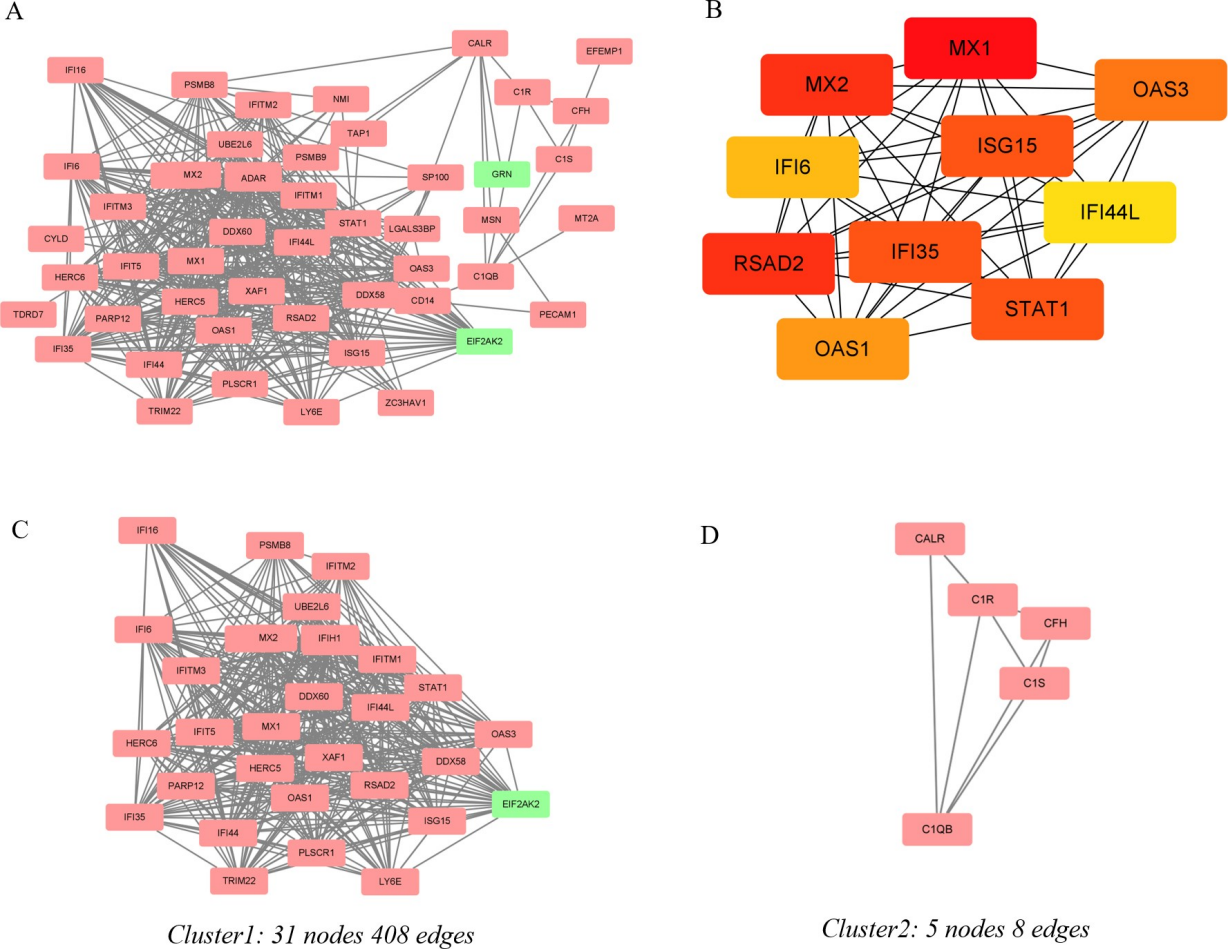
PPI network of DEGs and two cluster modules extracted by MCODE. (A) The interaction network among proteins coded by DEGs. Each node represents a protein, whereas each edge represents one protein–protein association. The red rectangles represent the upregulated gene, and the green ones represent the downregulated gene. (B) A network of the top 10 hub genes from the PPI network. Node color reflects the degree of connectivity. Red represents a higher degree, and yellow represents a lower degree. Two cluster modules extracted by MCODE. Cluster 1(C) had the highest cluster score (MCODE score = 27.2), followed by cluster 2 (D) (MCODE score = 4).

After importing the overlapping genes into STRING, the PPI network for the 63 DEGs was constructed (Fig 4A). The network provides information on the interactions among different genes, and it helps to mine the underlying mechanisms. Based on the information in the STRING database and Cytoscape, PPI relationships were obtained, and the hub genes in the network were identified (Fig 4B). The top 10 hub genes with high degrees were detected, including myxovirus (influenza virus) resistance 1 (MX1), myxovirus (influenza virus) resistance 2 (MX2), radical S-adenosyl methionine domain containing 2 (RSAD2), STAT1, IFN-induced protein 35 (IFI35), ISG15 ubiquitin-like modifier (ISG15), 2′-5′-oligoadenylate synthetase 3 (OAS3), 2′-5′-oligoadenylate synthetase 1 (OAS1), IFN alpha-inducible protein 6 (IFI6), and IFN-induced protein 44-like (IFI44L) (Table 2). All the top 10 hub genes were upregulated. MCODE was applied to identify the most significant module. Two modules matching the condition that MCODE score ≥4 are demonstrated in Fig 4C and 4D. The red rectangles represent the upregulated genes, and the green ones represent the downregulated genes. Cluster-1 (MCODE score = 27.2) has 31 nodes and 408 edges (Fig 4C). Cluster-2 (MCODE score = 4) has 5 nodes and 8 edges (Fig 4D).

**Table 2:** The sensitivity and specificity of the 10 hub genes in detecting DM.

Rank	Gene symbol	The name of protein	Regulation	Sensitivity (%)	Specificity (%)	AUC(95% CI)	Cut-off value
**1**	MX1	myxovirus (influenza virus) resistance 1	UP	100	90.3	0.947 (0.884–1.000)	1959.4
**2**	MX2	myxovirus (influenza virus) resistance 2	UP	100	93.5	0.991 (0.973–1.000)	1192.9
**3**	RSAD2	radical S-adenosyl methionine domain containing 2	UP	100	90.3	0.965 (0.920–1.000)	1043.2
**4**	STAT1	signal transducer and activator of transcription 1	UP	100	100	1.000 (1.000–1.000)	5522.5
**5**	IFI35	interferon-induced protein 35	UP	100	87.1	0.977 (0.943–1.000)	1024.25
**6**	ISG15	ISG15 ubiquitin-like modifier	UP	100	90.3	0.968 (0.921–1.000)	2554.37
**7**	OAS3	2’-5’-oligoadenylate synthetase 3	UP	92.9	100	0.991 (0.971–1.000)	1072.45
**8**	OAS1	2’-5’-oligoadenylate synthetase 1	UP	100	90.3	0.965 (0.917–1.000)	387.8
**9**	IFI6	interferon, alpha-inducible protein 6	UP	100	83.9	0.949 (0.888–1.000)	1368.372
**10**	IFI44L	interferon-induced protein 44-like	UP	100	90.3	0.965 (0.919–1.000)	1555.8

### Verification of the identified ten hub genes by datasets GSE1551 and GSE11971

**Fig 5:**
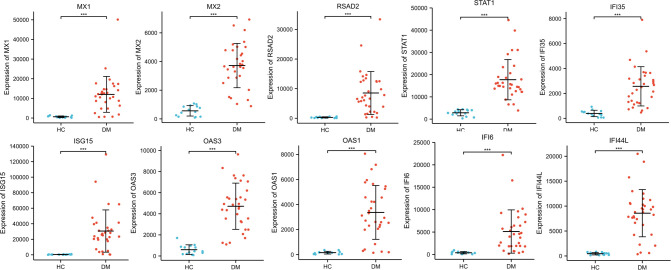
Verification of the 10 hub genes by GSE1551 and GSE11971 datasets. ***: P < 0.001, all hub genes are upregulated in DM samples with significance.

The GSE1551 and GSE11971 both from the GPL96 platform, which included 32 DM samples and 14 HC samples, were used to verify the expression of 10 identified hub genes. The R package ggplot2 was applied to draw boxplots. In addition, the R package ggpubr was used to perform Student’s t test statistical analysis. Based on the results, the mRNA expression levels of the 10 hub genes in the DM samples were significantly increased (P < 0.01, Fig 5).

### ROC curves of the ten hub genes in DM samples

**Fig 6:**
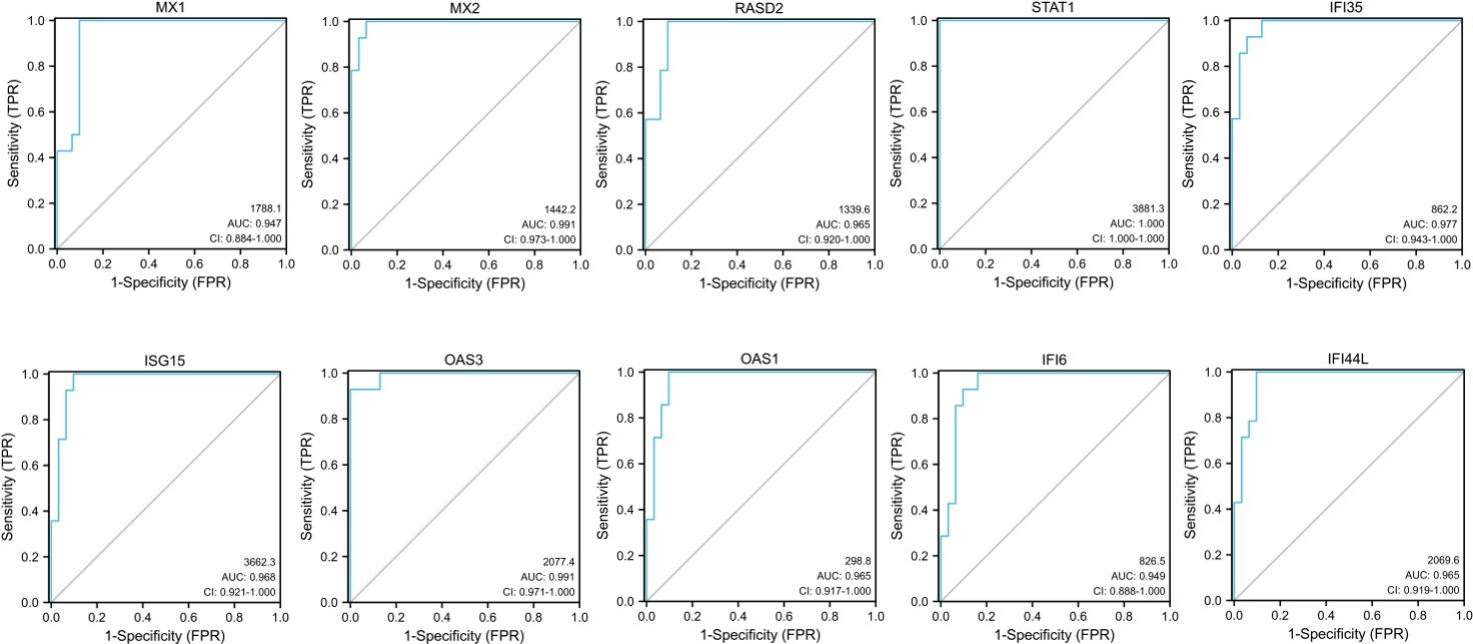
ROC curves of the 10 hub genes constructed by GSE1551 and GSE11971 datasets. The 95% confidence interval (CI), AUC, and predictive variable of each hub genes are shown in the figure.

GraphPad PRISM 7 was used to analyze the expression profiles of 10 hub genes and draw the ROC curves. AUC is an effective and combined measure of sensitivity and specificity for assessing inherent validity of a diagnostic test [21]. The different expression levels of the hub genes were searched in the Series matrix file of GSE1551 and GSE11971. The exact values are presented in the table and imported into the software. The sensitivity, specificity, cut-off value, and AUC are calculated by GraphPad PRISM 7 and listed in Table 2. When the AUC is over 0.8, the sensitivity and specificity are over 80%, indicating that the biomarker has good diagnostic accuracy. STAT1 has the highest diagnostic value (AUC: 1). The AUC of other genes are as follows: MX1 (AUC:0.947), MX2 (AUC:0.911), RSAD2 (AUC:0.965), IFI35 (AUC:0.977), ISG15 (AUC:0.968), OAS3 (AUC:0.991), OAS1 (AUC:0.965), IFI6 (AUC:0.949), and IFI44L (AUC:0.965) (Fig 6). Given their good diagnostic performance, we hypothesize that the identified hub genes may be potential biomarkers for the diagnosis of DM based on our present samples.

## Discussion

In this study, a series of bioinformatic analysis identified 10 hub genes (MX1, MX2, RSAD2, STAT1, IFI35, ISG15, OAS3, OAS1, IFI6, and IFI44L) between skeletal muscle biopsy of DM and HC samples based on the gene expression profiles obtained from GSE48280 and GSE5370 datasets. The results were validated by GSE1551 and GSE1171 datasets. Based on the results of ROC-AUC analysis, the identified 10 hub genes have good diagnostic performance. Meanwhile, we investigated the biological functions of these common DEGs. GO and KEGG analyses revealed that these DEGs were significantly associated with changes in response to virus and type I IFN signaling pathway.

MX1 and MX2 are IFN‐inducible myxovirus dynamin‐like GTPase, which are widely found in many mammals, whereas MX2 is only localized to the nucleus by a basic nuclear localization signal, which is required for antiviral activity [22]. Most of the previous studies have focused on the role of MX2 gene as an IFN‐induced restriction factor of different viruses [23, 24]. According to our result, MX1 and MX2 are highly expressed in muscle tissue of DM patients. The first case report regarding myxovirus and myositis was in 1979. The researchers isolated influenza A and B viruses in a patient with acute polymyositis [25]. Recently, the sarcoplasmic expression of MX1 has been demonstrated as a hallmark of DM [26, 27]. These findings can be considered as a supplement of the role of IFN pathway activation in patients with DM. MDA5, a specific antibody target of DM, plays a vital role in innate immunity by recognizing viral RNA in the cytoplasm and transmitting an induction signal downstream for type-1 IFN production [28]. These data indicate the potential involvement of a viral infection as a trigger moment for this systemic autoimmune disease. However, the relationship between the MX family and DM has not been fully revealed. Therefore, the exact role of the MX family in pathogenesis of DM may need to be further investigated.

STAT1, identified as the mediator of the cellular response to IFN, is activated by cytokine receptors via kinases of the JAK family [29]. Similar to our finding, recent studies have shown that the expression of STAT1 is significantly increased in the muscle tissue of patients with DM than in the normal control group [30]. This year, Julie J. Paik et al. proved that tofacitinib, a JAK-STAT inhibitor, was clinically effective in treating DM [31]. ISG15, a type I IFN-inducible protein, is highly upregulated in muscle, blood, and skin of patients with DM [32, 33]. ISG15 production has been well characterized from type I IFN stimulation. However, what drives this production in DM is uncertain. In our study, ISG15 is considered as a good biomarker in diagnosing DM, and the AUC is up to 0.968. OAS proteins are double-stranded RNA-activated enzymes, which are induced by IFNs. The OAS family consists of four members, namely, OAS1, OAS2, OAS3, and OAS-like protein (OASL) [34]. Similar to our result, a high expression level of the OAS3 is detected in muscle biopsy of DM patients according to Musumeci’s study in 2018.They concluded that the activation of IFN pathway could result in the upregulation of OAS3. Therefore, the IFN pathway could be the key pathway in the pathogenesis of DM.

Meanwhile, GO and KEGG analyses revealed that DEGs in our analysis were significantly associated with changes in response to virus and type I IFN signaling pathway. Viral antigens might play a triggering role in the onset of DM. This hypothesis is in accordance with numerous clinical observations suggesting a possible link between myopathies and infectious triggering agents [35]. With regard to the type I IFN signaling pathway, it consists of an important group of cytokines that are produced by innate immune cells, acting on adaptive immune cells. Overexpressed type I IFN-inducible transcripts and activated type I IFN signatures have been reported more than once in patients with DM [36, 37]. Consistent with previous studies, we discovered that DEGs were enriched in this signaling pathway. Hence, inhibiting type I IFN signatures would be a new treatment target for DM.

In the interpretation of our results, the following limitations require careful discussion. On the one hand, we only focused on the hub genes, whereas the other DEGs, which may also have a diagnostic and therapeutic effect, were ignored. On the other hand, the identified genes and their pathways were not confirmed through in vitro assays or in vivo models. Therefore, precisely designed studies are necessary to verify these potential genes.

Our results could be further used to predict DM-related noncoding-RNAs (ncRNAs), which may be the future direction of our work. MicroRNA is a class of endogenous, small, evolutionarily conserved ncRNAs, normally act as a negative regulator of mRNAs. Several useful databases and tools could be applied for potential microRNA-disease associations [38–41]. MicroRNA can interact with long non-coding RNAs (lncRNAs) and circular RNAs (circRNAs) in many diseases. Several well-designed algorithms can be used to discover the relationship among ncRNAs [42–45]. Given that the competing endogenous RNA (ceRNA) has not been established for DM, using these valuable tools, we hope to construct the ceRNA network in our future work.

## Conclusion

In conclusion, based on integrated bioinformatic analyses, we identified differences in biological functions in DM compared with HC samples. In particular, our work identified 10 valuable genes in muscle tissue as potential biomarkers for the diagnosis of patients with DM. The identified hub genes all have good diagnostic performance, which may serve as new biomarkers for DM. Drugs targeting these hub genes would become new treatment options for this high-mortality disease. Thus, this analysis may guide future experimental research and clinical transformation.
